# The harmful effects of legislative restrictions on gender affirming hormone therapy in Florida and Missouri: Challenges for patients and providers

**DOI:** 10.1371/journal.pone.0329075

**Published:** 2025-07-23

**Authors:** Lauren Porsch-Ortega, Sara Birnel Henderson

**Affiliations:** Research and Evaluation Department, Planned Parenthood Federation of America, New York, New York, United States of America; McMaster University, CANADA

## Abstract

Among the recent surge of legislative restrictions on the provision of gender affirming care to youth in the United States, some legislation, such as bills passed in 2023 in Florida and Missouri, have impacted the provision of care to adults. We sought to understand the impacts of these restrictions on both adult patients and health care providers seeking and providing gender affirming hormone therapy. We conducted semi-structured qualitative interviews with 21 patients and seven health care providers and staff from Planned Parenthood health centers in these two states and identified key themes related to their experiences of accessing and providing gender affirming hormone therapy before and after the implementation of the restrictions on care. Main themes emerging from interviews with both patients and providers/staff included disruptions to accessing or providing care after the restrictions were implemented, their range of reactions to learning about the restrictions, the negative impacts of the restrictions on transgender and gender diverse (TGD) people’s access to health care and providers’ provision of care, and what they want policymakers to know about the impact of the restrictions. Providers and staff also discussed the internal rewards of providing gender affirming hormone therapy, and patients shared the negative consequences of the restrictions on their lives and well-being. Our findings suggest that legislative restrictions on gender affirming care cause psychological, physical, and financial harm to TGD individuals by creating conditions that impose lapses in care and contribute to a hostile environment for TGD people.

## Introduction

Gender affirming hormone therapy (GAHT) has been demonstrated to improve the health and quality of life of transgender and gender expansive (TGD) individuals [1–5]. The safety of GAHT has been well-established, as evidenced by a large-scale systematic review including studies from 2015 to 2021 that reflect current medical regimens used for GAHT [6]. Nevertheless, TGD people face mounting barriers to accessing this form of medically-necessary health care.

Two recent scoping reviews of the literature on barriers and facilitators to accessing gender-affirming health care illuminated the extensive barriers faced by TGD individuals trying to obtain GAHT [7,8]. One of these reviews also examined barriers and facilitators for clinicians in providing this care [7]. Key barriers faced by TGD people included financial and insurance barriers, difficulty finding a provider of GAHT, and lengthy assessment processes required by providers prior to starting hormones [7,8]. Health care providers also face insurance-related barriers to providing GAHT, as well as a lack of training opportunities to provide GAHT [7]. A large body of existing research has also documented that some TGD individuals seek hormones outside of the traditional medical system when unable to access them from a licensed provider [9–20].

Facilitators to accessing care for patients included the use of an informed consent model of care (i.e., when patients are able to begin care following an assessment for medical eligibility and a discussion of the risks, benefits, and alternatives to care, without burdensome universally required clearance letters from mental health care providers) [7,21]. In addition, being able to access care from front-line health care providers such as primary care providers and sexual and reproductive health clinics, rather than specialists only, facilitated TGD individuals’ access to care [22,23]. Telemedicine has also been found to be a facilitator to care for both patients and providers [7].

**Fig 1:**
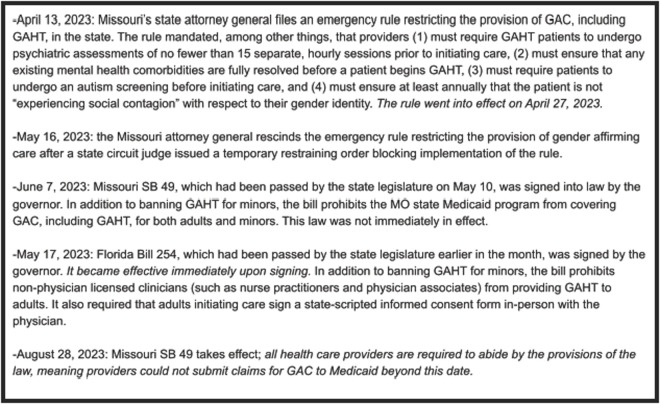
Timeline of regulatory and legislative actions affecting GAHT in Florida and Missouri, April to August 2023.

**Fig 2:**
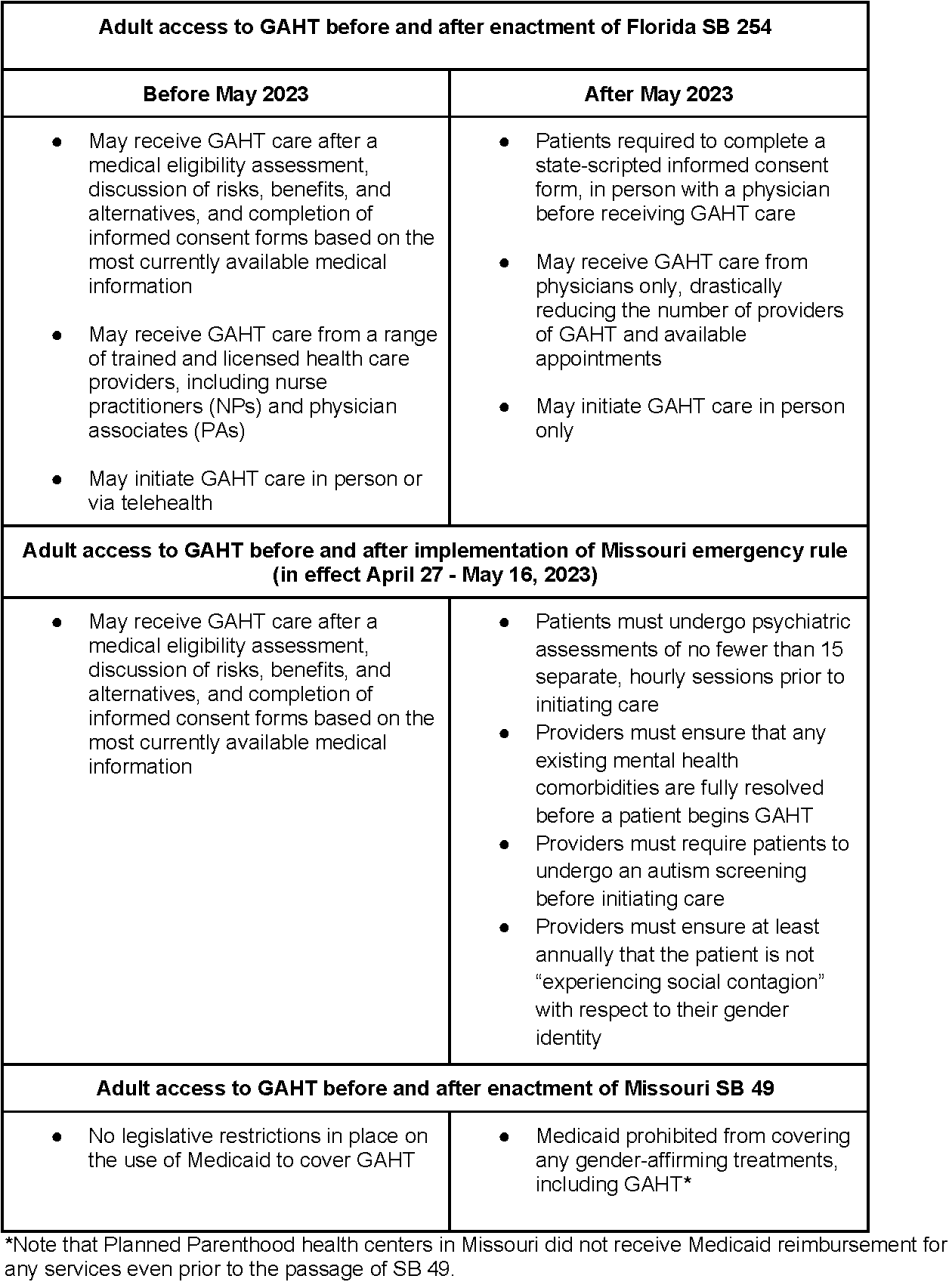
Summary of requirements for providing GAHT to adults in Florida and Missouri before and after the 2023 regulatory and legislative changes.

In recent years, there has been a spate of new state-level legislation in the U.S. seeking to limit or restrict the provision of gender affirming care (GAC), including GAHT [24–26]. Individual states in the U.S. have the ability pass laws affecting the provision of health care in their own states, sometimes resulting certain types of care being legal in some states, but not in others. Although many of the recently-passed GAC-related bills focus on the provision of care to minors, some, such as legislation implemented in Florida and Missouri in 2023, include restrictions that impact adults. On April 13, 2023, the Missouri state attorney general issued an emergency rule requiring multiple lengthy pre-screening requirements for both minors and adults seeking to access GAHT, which went into effect on April 27, 2023. This rule was ultimately rescinded on May 16, 2023, after the Missouri state legislature passed SB 49 on May 10, which prohibited the provision of GAC to minors and forbade the use of Medicaid funds for GAC, including for adults [27]. Also in May 2023, the Florida state legislature enacted SB 254, which prohibited the provision of GAC to minors altogether. SB 254 also stipulated that only physicians could provide GAHT to patients of any age (preventing licensed clinicians such as nurse practitioners and physician associates from providing this care), and that patients must present for care in-person to review and sign mandated state-scripted consent forms with the physician present [28] (see Fig 1 for a timeline of these regulatory and legislative actions and Fig 2 for a summary of requirements for providing GAHT before and after the changes). While banning care for minors outright, both pieces of legislation had the potential to drastically curtail care for adults. In the present study, we sought to better understand how these new restrictions affected TGD adults receiving GAHT, as well as the impact on the providers and staff who cared for these patients.

## Methods

We conducted a qualitative interview study with patients, providers, and staff from two Planned Parenthood affiliates, one in Florida, and one in Missouri. We assembled a group of study advisors, made up of Planned Parenthood patients who identified as transgender or gender expansive, to provide input on the design of the study and assist us with interpretation of the results. The study team met with the study advisors twice during the course of the study – once prior to beginning data collection, and a second time after the transcripts had been coded, to discuss preliminary findings. Prior to beginning study activities, the protocol received a determination of exemption from IRB oversight by the Advarra Institutional Review Board.

### Recruitment and data collection

SB 254 (Florida) and SB 49 (Missouri) went into full effect in May 2023 and August 2023, respectively. All health care providers were required to be in compliance with the new laws as of those dates. We began participant recruitment on October 1, 2023 and continued through June 30, 2024. We used purposive sampling to recruit patients who had received GAHT, and providers (e.g., physicians, nurse practitioners) or staff (e.g., patient navigators, patient care associates) who had worked on the delivery of GAHT services, at the participating Planned Parenthood affiliates. Health center staff at study sites distributed paper and/or electronic study fliers to patients receiving GAHT services, during in-person appointments, or via their secure electronic medical record portal. The study team distributed interview invitations by email among providers and staff through a central contact at each affiliate. Patients could opt-in to learning more about the study by filling out an online eligibility screening form linked by QR code on the flyer. All patients who screened eligible were contacted by researchers to schedule an interview. If the patient did not reply to the request for an interview, the next eligible patient was contacted until we met our recruitment target and preliminary review of the transcripts indicated saturation was reached. For provider and staff recruitment, a central contact at the study sites sent an invitation email to all staff involved in GAHT care at those sites, and staff/providers could opt in to the study by emailing the researchers. All of the staff/providers who contacted the research team met eligibility criteria and were included in the study. For patients, the eligibility criteria were age 18 years or older, and that the patient had attended an appointment for GAHT (defined as screening for, initiation, or monitoring for hormone therapy) at one of the participating Planned Parenthood affiliates in the last 12 months. For providers and staff, the eligibility criteria were having provided care to GAHT patients at one of the participating Planned Parenthood affiliates for at least six months prior to the implementation of legislative restrictions on GAC in their state.

The two authors (LPO and SBH) collected data via in-depth interviews over videoconference. We followed a semi-structured interview guide that included questions on participants’ history taking GAHT, experiences accessing care before and after the legislative restrictions were implemented, and how the implementation of the restrictions affected them. Participants provided verbal informed consent prior to the initiation of the interview, which was witnessed and documented by the interviewers after reviewing the potential risks, benefits, and alternatives of study participation. All participants chose pseudonyms for confidentiality. Twenty-one patients and seven providers or staff met study eligibility criteria and completed interviews with one of the two authors. The authors transcribed the interviews, which averaged 40 minutes in length, verbatim. Participants received a $50 gift card after completion of the interview.

### Data analysis

We used Dedoose software (ver. 9.2.007, 2024, Los Angeles, CA) to code all interview transcripts. Employing a combination of deductive and inductive coding, we engaged in an iterative process to develop a coding scheme (code book) and identify major and minor themes emerging from the interview data. Based on prior research documenting negative outcomes to TGD people when they faced difficulty accessing GAHT [7–20], we began with a broad hypothesis that the new legislative restrictions on care would have detrimental effects on TGD patients. We also looked for the emergence of divergent themes in the data. All transcripts were coded independently by both authors, and then re-coded after the authors met to discuss discrepancies in coding and achieve consensus.

## Results

### Provider and staff results

We interviewed seven providers and staff, including one physician, three nurse practitioners, and three patient care associates/patient navigators. The experiences shared by providers and staff were grouped into five major thematic areas: 1) the differences in providing GAHT before and after restrictions, 2) the rewards of providing GAHT care, 3) their reactions to the restrictions, 4) the negative impacts of the legislation on them and their patients, and 5) what they want policymakers to know.

### Differences in providing GAHT before and after the restrictions

Providers faced several challenges in providing GAHT care even prior to the implementation of legislative restrictions, including few opportunities for training in GAHT during their formal schooling or post-graduate training. All of the providers we interviewed explained that they had received this training through their employer or had sought out training independently. Providers also described several facilitators to providing GAHT care prior to the implementation of legislative restrictions. These included providing care via telehealth, support from organizational leadership to provide GAHT, providing care through the informed consent model, and building community partnerships with transgender community organizations that provided additional health and wellness resources for patients (i.e., “wrap around” services). As one clinician explained:

*“There are a lot of rural folks that are needing this kind of care and not able to get out to a big metro area. So we started providing telehealth care, the agency that we partnered with - the community agency - has also opened up space for our clients to come in and pick up their supplies or get their lab work drawn….And a big thing that we notice is a lot of folks don’t want to come into the health center because they’ve had negative experiences. And so, our plan was, if we could kind of ease them through telehealth and through a group of people that they know already then maybe we could meet the needs they have today, but then [later] also do preventative care for you.” - Amy, nurse practitioner*

### Rewards of providing GAHT care

Providers and staff discussed the personal rewards of providing GAHT, which often stemmed from seeing the positive effects of GAHT on patients, and patients’ gratitude for the care they received. Providers expressed that their experiences providing GAHT often affirmed why they chose a career in health care:

*“I mean you could kind of sometimes see just relief on patients’ faces. They’ve been waiting for this for a long time. It’s not like anybody decides yesterday that they’re gonna start hormones; it’s usually a very long, thought-out decision and usually with a lot of soul searching to get there. So that’s really rewarding, and then I also really loved the first three month visit back when people had been on meds and are starting to see changes and usually are really very happy. …That’s really rewarding and part of the reason I think we go into health care and medicine.” - Lucy, physician*

### Provider and staff reactions to restrictions

When first learning about the legislative restrictions on GAHT, providers and staff experienced deep worry and concern about the impact on patients. Many providers and staff expressed feelings that demonstrated a sense of moral injury, defined in the psychology literature as “the strong cognitive and emotional response that can occur following events that violate a person’s moral or ethical code” [29]. In being effectively barred from providing care that both patients and providers deemed medically necessary, providers and staff experienced extreme distress. When explaining the effect of the “physician-only” requirement in the new Florida law, one staff member explained:

*“It was very wrecking because there’s only so many providers, as in doctors, for our affiliates. Knowing that we have thousands of patients that this would affect, it was a very hard time because we had to pause care. And having to make those phone calls was extremely difficult…patients [who] have been on these prescriptions for years…to tell them we can’t refill your prescription right now because of a new law… it was heartbreaking. It was very trauma-filled. It was very hard to experience that - hearing them cry. It was rough.” - Kelly, staff*

Between learning about the impending restrictions and the date that the restrictions took effect, providers and their teams worked creatively to provide care. They tried to get as many patients in for appointments as possible in the intervening period. They also extended prescriptions for as long as they safely could.

*“So what we did do is, because of course, we didn’t know when exactly the change was going to happen. We began to prepare for it happening by writing the prescription for as long as we could write it while being safe. So if we’ve seen you, if your labs have been good…for our feminizing patients, we would give them a year. And for our masculinizing patients, we could only give six months at a time. So if we felt safely we could do that - there was nothing unusual or abnormal in their history, we would write the prescription for as long as we could.” – Martha, nurse practitioner*

### Negative impacts of restrictions on provision of care

Providers and staff also discussed the challenging experiences they had providing care after the new restrictions became effective, and how the restrictions negatively impacted them and their patients. For example, their health centers received an influx of calls and messages from fearful and confused patients. In Missouri, the confusion and fear was compounded by the rapid succession of the issuance of the emergency rule, the rescinding of the emergency rule, and the intervening passage of SB 49. A physician we interviewed stated *“I mean when I think about our nurses - all the calls when all this stuff was going on, they got a huge volume of phone calls with people just freaking out and rightly so.”*

Affiliates paused care to adapt their care models to comply with the new law. When services resumed, there were fewer appointments available and longer wait times for patients. This was compounded by a lack of alternative providers for patients to see. One nurse practitioner shared, *“we tried to put together a list of other providers, and there weren’t any after the new law because nobody wanted to touch it.”*

Providers and staff described multiple patient-level impacts, including patients having to travel long distances to access care and patients considering moving out of state. They also heard from patients looking into what they described as “do-it-yourself (DIY)” methods of accessing and taking hormones, such as trying to obtain them outside of the traditional medical system and/or taking their medications at different dosages than they were prescribed. A staff member explained:

*“Some patients were saying they were cutting their prescriptions in half. They were finding old vials that they would normally just toss they were keeping them. It was scary. They were saying I guess pharmacies [had] stopped…they weren’t giving them their needles anymore. So they were having to buy them online a lot and things going to different countries and getting their medications. Yeah, it’s like they were doing anything that they could to keep their normal life going, and it’s scary because you don’t know the prescription’s real. You don’t know what you’re really putting in your body.” - Kelly, staff*

### What they want policymakers to know

Providers and staff wanted to share with policymakers that GAHT is medically-necessary care for their TGD patients. Just like treatment for any other medical condition, GAHT is often the proper treatment for patients with gender dysphoria. One provider explained:

*“I would say that this is not elective care. So this is not like getting a breast augmentation or a Lasix or something, like this is actually medically required care. So if somebody comes to me and expresses dysphoria with their sex assigned at birth, then the right treatment is to help them with whatever way that is right for them. So if that’s hormones or surgery or whatever like it should not be considered something extra. It should be considered the same as if somebody comes to me for their high blood pressure and needs a blood pressure medicine, or somebody comes from me for their ovarian mass and needs their ovary taken out, that this is regular health care. It’s just treating a different condition.” - Lucy, physician*

## Patient results

Like with providers and staff, we identified five major thematic areas from the experiences shared by the twenty-one patients we interviewed. These include 1) patients’ baseline experiences accessing care prior to the restrictions, 2) patients’ initial reaction to the restrictions, 3) the difficulties experienced accessing care after the implementation of the restrictions, 4) the consequences of difficulties accessing care, and 5) what they want policymakers to know.

### Baseline experiences accessing care

Prior to the implementation of restrictions on GAC, participants expressed having a range of experiences accessing care, from having multiple difficulties getting care to accessing it with relative ease. The most frequently mentioned barriers to accessing care before the restrictions were the limited number of providers who prescribed GAHT, financial and insurance difficulties, and lack of family support. One patient explained how lack of family support exacerbated her financial barriers to care:

*“I’ve looked into it before and as far as I think the government sponsored health insurance, I’m just not eligible to get it for myself. And so I’d have to go through my parents’ insurance, and they don’t have it. But even if they did enroll, they said that they wouldn’t allow me to use any of it towards gender affirming care.” - Avery, patient*

In contrast, the most commonly discussed facilitators to accessing care included telehealth, providers’ use of an informed consent model of care (as opposed to universally requiring a lengthy mental health clearance process), and having peer or family support.

*“I started the process by making an appointment… for informed consent. It makes it much more streamlined. So basically to [review] the list of all the possible side effects…but it did go into deeper information [about what happens when you start] HRT. Yeah, that you’ll grow breasts. So what we’re hoping for should be hair changes, all that stuff. [To make sure] that I understood what I was doing…The [informed consent model] helped because it means I didn’t have to go through a back-and-forth saying we have to make sure you’re totally trans. That was very useful for me. Because…like how much more do you want me to prove I’m trans?” – Emily, patient*

Participants also discussed the profound positive effects that having access to GAHT had on their well-being, especially their mental health:

*“As far as my life goes before receiving hormones, I would suffer from suicidal ideations frequently as part of just the dysphoria, and once I started receiving hormones people started pointing out that I was smiling more… I was actually enjoying life at that point…and everything kind of took an upturn.” - Jen, patient*

### Another patient shared:

***“*** *I was on antidepressants before [starting GAHT], and after about two months [being on GAHT], I was able to come off of them. I felt better than I had even when I was on them. I also have agoraphobia and I found that after transitioning [I could] convince myself to work on that and actually leave the house. So I found my symptoms of anxiety lessen a little bit.” - Mike, patient*

### Patients’ initial reactions to the restrictions

Patients described having multiple initial reactions to learning about the new restrictions, including fear, worry, shock, and uncertainty. As one patient shared, *“it was a rough time because… I had the anxiety of what happens if that [bill] passes, what happens if it’s literally illegal to be myself.”* Some patients explained that although they were upset by the restrictions, they were not surprised by them, because they had been following the explosion of anti-trans legislation in their state legislatures:

*“I actually found out about it far before it passed. There were a trio of bills that I heard about…because they were extremely transphobic and extremely dangerous and I literally had the public page on the state’s website that shows the status of any given bill…And I would check it almost daily to see what the status was, …could we dodge this bullet or not? And I could literally see it in slow motion coming and that wasn’t great for my mental health.” -Kelsey, patient*

Many patients scrambled to make appointments prior to the new restrictions becoming effective. One patient said: *“I rushed [to make an appointment] when I realized that there would be a ban, because I have other mental health problems and knowing that [GAHT] was about to be beyond my reach was devastating.”*

### Patients’ difficulties accessing care after the implementation of the restrictions

After the implementation of the restrictions on GAC, patients experienced lapses in care, often due to lack of available appointments and a lack of alternative providers. A patient from Florida explained:

*“Sometime in the late summer, I took my last dose and I couldn’t get it renewed because of [the restrictions]...The system was completely bottlenecked. There were very limited doctors with appointments…there was a massive number of patients just waiting for an appointment. So from around July [when] I took my last dose to late November I did not have any testosterone. My prescription was completely paused and on hold.” – Mike, patient*

In accordance with the new law, Florida patients were required to have an in-clinic appointment with a physician to sign a state-mandated consent form before beginning or resuming care. When they were able to access care, patients faced additional burdens, including long-distance travel and/or interstate travel. Patients who were able to get in-state appointments described having to travel several hours further from their homes in order to see a physician. Another patient from Florida shared that they resorted to traveling out-of-state: *“But when I was starting to get desperate, I did try to fly…I had an appointment with Planned Parenthood in [distant state where GAC was not legislatively restricted]. I did try to travel out of state to try to get a three-month prescription of my hormones to…carry me over.”*

With regard to the state-scripted consent form, many described it as being full of misinformation and demeaning:

*“Signing all those forms is not fun and the MD was sitting there and said you have to initial every single line and so I went through all that… It was very dehumanizing to have to go through all that and basically be told that both the medical profession’s knowledge was inadequate and that my knowledge and research was inadequate to where the state was able to make the decision for me.” – Matthew, patient*

### Consequences of patients’ difficulties accessing care

Patients described significant consequences of their inability to access care. Many experienced negative physical and emotional effects from sudden forced detransition due to being cut off from GAHT. One patient shared:

*“So I knew I was gonna be off my hormones for a bit [and] I was starting to get desperate admittedly. Because being forcibly detransitioned, it’s not fun. The physical impacts of the shift is horrible and I had to kind of go about life and work and act like, it honestly kind of felt like I was trapped in a burning building and the doors were chained shut and having to go about life as if everything was okay and I wasn’t a physical and emotional wreck for several months, was tough.” Kelsey, patient*

In addition, several patients discussed ways that they tried to ensure they could continue to access their medications, including rationing or sharing medications:

*“I definitely started rationing my medication thinking about making sure I save some stuff…yeah, like I lowered my dose because…the medication, it says single-use vial…on the box, I was just using one time and then throwing it away, but there’s leftover. So once I heard that [the restriction] was happening, I started to save my vials so that was my way of rationing.” - Mathias, patient*

Other patients shared that they had begun looking into “do-it-yourself” (or “DIY”) methods of accessing hormones, or trying to obtain them outside of the traditional medical system:

*“I very much looked into DIY. I looked at several websites and I did a bunch of research into DIY. Which I know is risky and expensive but it was either that or go without my medication that I need….I don’t love that I was looking towards DIY. But if that’s what we had to do, that’s what we had to do. Because I know that I’m not the only one that had to at the very least look at that route, as there was a period of at least a month and a half, two months that Planned Parenthood just didn’t have appointments, they weren’t doing GAC. So a lot of trans Floridians just couldn’t access the care we needed.” - Morgan, patient*

### What patients want policymakers to know

Like providers, patients want policymakers to know that GAC is medically necessary for them and not elective care. They also hoped that policymakers would empathize with trans and nonbinary individuals and understand the suffering these restrictions cause, both directly in cutting off access to care, and indirectly in contributing to an environment of bigotry towards trans people. One patient shared:

*“I mean, I think the thing is that it is medically necessary… It’s not something that’s purely cosmetic or purely just a choice. This is something that’s drastically impacted my life for the better…. I’m happier every day. I feel better every day. I feel stronger and I think having policymakers [who are] not medical professionals trying to take that away and restrict it… it’s a really negative thing, and it hurts all of their constituents, and it doesn’t really benefit anybody.” - Avery B, patient*

Another patient described the personal harm he has experienced as a result of these laws:

*“I don’t feel like an equal citizen. And I haven’t for a long time. Even before this ban, and now that it is here, America has made it very clear that they do not see trans people as human beings worthy of equal rights….I feel like I’m being punished for living my life in the only way that I can. And I’ve tried very hard to not be trans and it doesn’t work. I’m so afraid. Because I have noticed people become so much more violent, who used to be middle-of-the-road. I’ve experienced harassment and abuse and potential harm in places, just…because I look different.” -Oren, patient*

## Discussion

Consistent with prior research, our findings demonstrate that access to GAHT has positive effects on the well-being of TGD individuals, and that this care can be lifesaving for many people who access it. Our participants overwhelmingly discussed the mental health benefits of receiving GAHT, such as reduced depression, anxiety, suicidal ideation, and body dysmorphia. Our findings also confirmed that the informed consent model and telehealth made GAHT more accessible for patients, reducing some of the main hurdles to care. Regarding barriers to care, the providers we interviewed confirmed that there is a lack of formalized training in gender affirming care in health professions training programs, which may result in fewer providers offering GAHT. Given this, legislative restrictions like Florida SB 254, which further limit the types of providers who can prescribe GAHT and require in-person visits with a physician, can have devastating effects on access to this care. The patients we interviewed highlighted the real-life impacts of these restrictions, including lapses in care, sudden forced detransition, and increased burdens in long distance travel and expense to obtain care.

Even prior to the wave of new legislative restrictions on GAC, the costs of care have been documented as a major barrier [7–8], and this care is not always covered by medical insurance, including Medicaid [30]. This makes legislative restrictions on Medicaid coverage for GAC (such as those implemented with Missouri SB 49) particularly harmful to people with low incomes, people of color, and young people who disproportionately rely on Medicaid for their health care coverage [31–32]. Our findings and those of prior research [9–20] demonstrate that TGD individuals may seek hormones outside of the traditional medical system when they are not able to access them from licensed providers.

While our findings aligned with previous research in many areas, some findings diverged from the existing literature. Previous studies have found that patients may feel pressured to conform to a binary gender presentation, and that providers may act as medical gatekeepers if patients’ gender identities and presentations do not align with their own beliefs about gender [33–35]. However, none of the participants in our study shared this experience. This difference in care experiences may be related to the use of the informed consent model at many Planned Parenthood centers, which centers patient autonomy and allows medically eligible patients to receive hormone therapy without multiple prior visits to, and clearance letters from, mental health professionals. Another novel finding was the role of family relationships in either facilitating or hindering access to care for adult patients. This is likely influenced by the fact that many young adults in the U.S., up to age 26, are covered by their parents’ health insurance and often live with their parents, relying on them for financial and practical support.

There are some limitations to this study that may affect the generalizability of the findings. Our current study was conducted exclusively with Planned Parenthood patients and providers; as such there may be different issues facing patients and providers working at/utilizing other health care organizations. In addition, the majority of the patients participating (57%) identified as white. While this reflects the patient demographics of the GAHT services at the participating health centers, we may have missed impacts of the legislative restrictions that uniquely affect TGD communities of color. Of note, in the interviews, many participants of all backgrounds expressed that their friends and community members from marginalized groups face more barriers and obstacles to care than they do.

Our findings have several implications for clinical practice and education, policy, and future research. It is imperative that health professional training programs incorporate routine training in GAHT care into their curricula. This education will equip a larger number of health care providers with the knowledge and skills to deliver GAHT care using current best practices. In the policy realm, legislators should work to remove onerous restrictions on the practice of GAC and ensure that it is covered by public and private insurance, which will improve access to this medically-necessary care. Finally, there is a need for future research focusing on the unique issues that TGD communities of color communities face in accessing GAHT to inform targeted strategies to reduce these barriers.

## References

[1] Murad MH, Elamin MB, Garcia MZ, Mullan RJ, Murad A, Erwin PJ, et al. Hormonal therapy and sex reassignment: a systematic review and meta-analysis of quality of life and psychosocial outcomes. Clin Endocrinol (Oxf). 2010;72(2):214–31. doi: 10.1111/j.1365-2265.2009.03625.x 19473181

[2] Amand CSt, Fitzgerald KM, Pardo ST, Babcock J. The Effects of Hormonal Gender Affirmation Treatment on Mental Health in Female-to-Male Transsexuals. Journal of Gay & Lesbian Mental Health. 2011;15(3):281–99. doi: 10.1080/19359705.2011.581195

[3] Colizzi M, Costa R, Todarello O. Transsexual patients’ psychiatric comorbidity and positive effect of cross-sex hormonal treatment on mental health: results from a longitudinal study. Psychoneuroendocrinology. 2014;39:65–73. doi: 10.1016/j.psyneuen.2013.09.029 24275005

[4] Fisher AD, Castellini G, Ristori J, Casale H, Cassioli E, Sensi C, et al. Cross-Sex Hormone Treatment and Psychobiological Changes in Transsexual Persons: Two-Year Follow-Up Data. J Clin Endocrinol Metab. 2016;101(11):4260–9. doi: 10.1210/jc.2016-1276 27700538

[5] Owen-Smith AA, Gerth J, Sineath RC, Barzilay J, Becerra-Culqui TA, Getahun D, et al. Association Between Gender Confirmation Treatments and Perceived Gender Congruence, Body Image Satisfaction, and Mental Health in a Cohort of Transgender Individuals. J Sex Med. 2018;15(4):591–600. doi: 10.1016/j.jsxm.2018.01.017 29463478 PMC5882508

[6] D’hoore L, T’Sjoen G. Gender-affirming hormone therapy: An updated literature review with an eye on the future. J Intern Med. 2022;291(5):574–92. doi: 10.1111/joim.13441 34982475

[7] Porsch-Ortega L, Athilat S, Kochhar S, McDonald M. Barriers and Facilitators to Accessing and Providing Gender Affirming Hormone Therapy: A Scoping Review. Transgender Health. 2024. doi: 10.1089/trgh.2023.0186

[8] Kearns S, Hardie P, O’Shea D, Neff K. Instruments used to assess gender-affirming healthcare access: A scoping review. PLoS One. 2024;19(6):e0298821. doi: 10.1371/journal.pone.0298821 38829881 PMC11146745

[9] de Haan G, Santos G-M, Arayasirikul S, Raymond HF. Non-Prescribed Hormone Use and Barriers to Care for Transgender Women in San Francisco. LGBT Health. 2015;2(4):313–23. doi: 10.1089/lgbt.2014.0128 26788772

[10] Lee JL, Huffman M, Rattray NA, Carnahan JL, Fortenberry JD, Fogel JM, et al. “I Don’t Want to Spend the Rest of my Life Only Going to a Gender Wellness Clinic”: Healthcare Experiences of Patients of a Comprehensive Transgender Clinic. J Gen Intern Med. 2022;37(13):3396–403. doi: 10.1007/s11606-022-07408-5 35112278 PMC8809217

[11] Linander I, Alm E, Hammarström A, Harryson L. Negotiating the (bio)medical gaze - Experiences of trans-specific healthcare in Sweden. Soc Sci Med. 2017;174:9–16. doi: 10.1016/j.socscimed.2016.11.030 27960120

[12] Glick JL, Andrinopoulos KM, Theall KP, Kendall C. “Tiptoeing Around the System”: Alternative Healthcare Navigation Among Gender Minorities in New Orleans. Transgend Health. 2018;3(1):118–26. doi: 10.1089/trgh.2018.0015 30014040 PMC6044177

[13] Mepham N, Bouman WP, Arcelus J, Hayter M, Wylie KR. People with gender dysphoria who self-prescribe cross-sex hormones: prevalence, sources, and side effects knowledge. J Sex Med. 2014;11(12):2995–3001. doi: 10.1111/jsm.12691 25213018

[14] Metastasio A, Negri A, Martinotti G, Corazza O. Transitioning Bodies. The Case of Self-Prescribing Sexual Hormones in Gender Affirmation in Individuals Attending Psychiatric Services. Brain Sci. 2018;8(5):88. doi: 10.3390/brainsci8050088 29757929 PMC5977079

[15] Regmi PR, van Teijlingen E, Neupane SR, Marahatta SB. Hormone use among Nepali transgender women: a qualitative study. BMJ Open. 2019;9(10):e030464. doi: 10.1136/bmjopen-2019-030464 31640998 PMC6830677

[16] Rotondi NK, Bauer GR, Scanlon K, Kaay M, Travers R, Travers A. Nonprescribed hormone use and self-performed surgeries: “do-it-yourself” transitions in transgender communities in Ontario, Canada. Am J Public Health. 2013;103(10):1830–6. doi: 10.2105/AJPH.2013.301348 23948009 PMC3780733

[17] Sanchez NF, Sanchez JP, Danoff A. Health care utilization, barriers to care, and hormone usage among male-to-female transgender persons in New York City. Am J Public Health. 2009;99(4):713–9. doi: 10.2105/AJPH.2007.132035 19150911 PMC2661470

[18] Singh Y, Aher A, Shaikh S, Mehta S, Robertson J, Chakrapani V. Gender Transition Services for Hijras and Other Male-to-Female Transgender People in India: Availability and Barriers to Access and Use. International Journal of Transgenderism. 2014;15(1):1–15. doi: 10.1080/15532739.2014.890559

[19] Smart BD, Mann-Jackson L, Alonzo J, Tanner AE, Garcia M, Refugio Aviles L, et al. Transgender women of color in the U.S. South: A qualitative study of social determinants of health and healthcare perspectives. Int J Transgend Health. 2020;23(1–2):164–77. doi: 10.1080/26895269.2020.1848691 35403118 PMC8986221

[20] Kennedy CE, Yeh PT, Byrne J, van der Merwe LLA, Ferguson L, Poteat T, et al. Self-administration of gender-affirming hormones: a systematic review of effectiveness, cost, and values and preferences of end-users and health workers. Sex Reprod Health Matters. 2021;29(3):2045066. doi: 10.1080/26410397.2022.2045066 35312467 PMC8942532

[21] Deutsch MB. Use of the Informed Consent Model in the Provision of Cross-Sex Hormone Therapy: A Survey of the Practices of Selected Clinics. International Journal of Transgenderism. 2012;13(3):140–6. doi: 10.1080/15532739.2011.675233

[22] Ingraham N, Fox L, Gonzalez AL, Riegelsberger A. “I just felt supported”: Transgender and non-binary patient perspectives on receiving transition-related healthcare in family planning clinics. PLoS One. 2022;17(7):e0271691. doi: 10.1371/journal.pone.0271691 35862408 PMC9302788

[23] Ker A, Fraser G, Lyons A, Stephenson C, Fleming T. Providing gender-affirming hormone therapy through primary care: service users. J Prim Health Care. 2020;12(1):72–8. doi: 10.1071/HC19040 32223853

[24] Williams Institute. Prohibiting Gender-Affirming Medical Care for Youth. 2023.

[25] Dawson L, Kates J. The proliferation of state actions limiting youth access to gender affirming care. https://www.kff.org/policy-watch/the-proliferation-of-state-actions-limiting-youth-access-to-gender-affirming-care/. 2024. 2024 February 8

[26] Seiler N, Spott A, Washington M, et al. Gender identity, health, and the law: An overview of key laws impacting the health of transgender and gender non-conforming people. St Louis Univ J Health Law Policy. 2023;16(22).

[27] Establishes the “Missouri Save Adolescents from Experimentation (SAFE) Act” and modifies provisions relating to public funding of certain gender transition procedures. https://www.senate.mo.gov/23info/BTS_Web/Bill.aspx?SessionType=R&BillID=44407

[28] Senate Bill 254 (2023). The Florida Senate. 2023. https://www.flsenate.gov/Session/Bill/2023/254

[29] Litz BT, Stein N, Delaney E, Lebowitz L, Nash WP, Silva C, et al. Moral injury and moral repair in war veterans: a preliminary model and intervention strategy. Clin Psychol Rev. 2009;29(8):695–706. doi: 10.1016/j.cpr.2009.07.003 19683376

[30] Movement Advancement Project. Health care/ Medicaid coverage of transgender-related care. https://www.lgbtmap.org/equality-maps/medicaid. 2023. 2024 August 20

[31] Pillai A, Hinton E, Rudowitz R. Medicaid Efforts to Address Racial Health Disparities. https://www.kff.org/medicaid/issue-brief/medicaid-efforts-to-address-racial-health-disparities/. 2024. 2024 August 20

[32] Coffey A, Adams G, Hahn H. Young People and Medical Assistance. Washington, D.C.: Urban Institute. 2021.

[33] MacKinnon KR, Grace D, Ng SL, Sicchia SR, Ross LE. “I don’t think they thought I was ready”: How pre-transition assessments create care inequities for trans people with complex mental health in Canada. International Journal of Mental Health. 2020;49(1):56–80. doi: 10.1080/00207411.2019.1711328

[34] Czimbalmos M, Rask S. (Dis)advantaged positions in accessing gender-affirming healthcare in Finland: an intersectional qualitative study of foreign-origin transgender people. BMC Health Serv Res. 2022;22(1):1287. doi: 10.1186/s12913-022-08654-3 36284312 PMC9597978

[35] Fraser G, Brady A, Wilson MS. “What if I’m not trans enough? What if I’m not man enough?”: Transgender young adults’ experiences of gender-affirming healthcare readiness assessments in Aotearoa New Zealand. Int J Transgend Health. 2021;22(4):454–67. doi: 10.1080/26895269.2021.1933669 37808530 PMC10553372

